# The m^6^A demethylase FTO regulates TNF-α expression in human macrophages following *Toxoplasma gondii* infection

**DOI:** 10.1371/journal.pntd.0013289

**Published:** 2025-07-15

**Authors:** Min Qin, Nan Gao, Jierui Sun, Shuqing Lin, Tingting Hu, Xinjian Liu, Rong Zhang, Yong Wang, Jingfan Qiu

**Affiliations:** 1 Key Laboratory for Pathogen Infection and Control of Jiangsu Province, Department of Pathogen Biology and Immunology, Nanjing Medical University, Nanjing, Jiangsu, China; 2 Department of Pathology, Children’s Hospital of Nanjing Medical University, Nanjing, Jiangsu, China; UNMC: University of Nebraska Medical Center, UNITED STATES OF AMERICA

## Abstract

*Toxoplasma gondii* (*T. gondii*) is an opportunistic parasite. After infection, macrophages finely regulate the immune response to restrict parasite proliferation. It is well-known that *N*^6^-methyladenosine (m^6^A) plays a critical role in fine-tuning gene expression. To investigate whether m^6^A modification is involved in regulating the anti-infection immune response in human macrophages against *T. gondii*, this study utilized *T. gondii* tachyzoites from the RH strain to infect human THP-1 macrophages. qPCR and ELISA results show that *T. gondii* infection mounted the expression of TNF-α, IL-1β, and IL-6. Transcriptomic data suggest that the infection of *T. gondii* induced differential gene expression in pathways associated with TNF signaling and cytokine-cytokine receptor interaction. Meanwhile, expression of m^6^A regulators were evaluated using qPCR and Western blotting. *T. gondii* infection increased the abundance of m^6^A demethylase FTO and methyltransferase WTAP. Joint analysis of RNA-seq and m^6^A-seq data was utilized for enriching differentially expressed genes (DEGs) with significantly altered m^6^A modifications. Intriguingly, following *T. gondii* infection, the m^6^A levels of DEGs associated with toxoplasmosis, TNF signaling pathway, and NF-κB signaling pathway were significantly different. The m^6^A-IP-qPCR assay further confirmed that *T. gondii* infection led to the decrease in the levels of m^6^A modification in the 3’UTR and 5’UTR regions of *TNF-α* mRNA. Knocking-down of FTO retarded the infection induced-decrease in the levels of m^6^A modification in *TNF-α* transcripts, accompanied by dampened immune response and uncontrolled *T. gondii* proliferation. Furthermore, the YTHDF2 RIP assay indicates that *T. gondii* infection remarkably weakened the binding of YTHDF2 to *TNF-α* mRNA, which could mount TNF-α expression by inhibiting the degradation of *TNF-α* mRNA. Overall, these findings suggest that m^6^A plays a role in the *T. gondii* infection-induced immune response in human macrophages, uncovering a new molecular mechanism for the regulation of TNF-α expression from an epitranscriptomic aspect.

## Author summary

One intriguing phenomenon in *T. gondii* infection and immunity is that the immune response induced by *T. gondii* is diverse in various hosts. Our previous research shows that in mouse macrophages, *T. gondii* microneme protein 3 (MIC3) activates TNF-α transcription via the TLR11/MyD88/NF-κB pathway. *T. gondii* infection can also induce TNF-α production in humans. However, human cells lack TLR11, leading us to predict that the mechanisms by which *T. gondii* regulates TNF-α expression in human macrophages differ from those in mouse macrophages. In this study, we found that *T. gondii* infection increased the abundance of m^6^A demethylase FTO. Joint analysis of RNA-seq and m^6^A-seq data indicates significant differences in the m^6^A levels of differentially expressed genes related to the TNF signaling pathway after *T. gondii* infection. Results of m^6^A-IP-qPCR assay further confirmed that *T. gondii* infection decreased the m^6^A modification levels in the 3’UTR and 5’UTR regions of *TNF-α* mRNA. Knocking-down FTO hindered the infection-induced decrease in m^6^A modification levels of *TNF-α* transcripts, accompanied by a dampened immune response and uncontrolled *T. gondii* proliferation. Additionally, *T. gondii* infection significantly weakened the binding of YTHDF2 to *TNF-α* mRNA, thereby promoting TNF-α expression by inhibiting *TNF-α* mRNA degradation. In sum, these findings explain why human macrophages lacking TLR11 can still express TNF-α after *T. gondii* infection and uncover a novel post-transcriptional regulation mechanism for TNF-α.

## Introduction

*Toxoplasma gondii* (*T. gondii*) is an important opportunistic parasite. In the healthy population*, T. gondii* infection does not cause obvious clinical symptoms [1,2]. However, infection occurs in the elderly and the immunocompromised individuals (such as AIDS patients, cancer patients and organ transplant recipients) could develop into acute toxoplasmosis, causing encephalitis, and even death [1,2]. Thus, the immune status of the host plays an important role in the development and outcome of *T. gondii* infection.

Macrophages play an indispensable role in resisting *T. gondii* infection and regulating immune homeostasis [3]. Among the inflammatory cytokines secreted by macrophages, tumor necrosis factor-α (TNF-α) has a highly pleiotropic effect in diverse cellular processes [4]. First of all, TNF-α is involved in controlling *T. gondii* infection. Blocking TNF-α may cause the latent infection of *T. gondii* to be reactivated, increasing the risk of acute toxoplasmosis [5]. Additionally, TNF-α could drive the conversion of tachyzoites to bradyzoites, resulting in the transition of toxoplasmosis from acute to chronic phase [6]. Furthermore, in spite of the protective role of TNF-α, excessive production of TNF-α can exacerbate inflammation and lead to immunopathology, contributing to the symptoms of acute toxoplasmosis, such as encephalitis [7]. Thus, it is of paramount importance to investigate the regulatory mechanisms of TNF-α after *T. gondii* infection.

In previous study, our research group focused on elucidating the transcriptional regulation mechanism of TNF-α. We demonstrated that MIC3 (microneme protein 3) activates *TNF-α* transcription via the TLR11/MyD88/NF-κB pathway in mouse macrophages [8]. In humans, *T. gondii* infection could also induce TNF-α production and human cells discriminate between viable and killed *T. gondii* tachyzoites [9–11]. However, human cells lack TLR11. Thus, we predicted that the mechanisms by which *T. gondii* regulates TNF-α expression in human macrophages are distinctly different from those in mouse macrophages.

It is well-known that post-transcriptional regulation helps cells respond more quickly to external stimuli than transcriptional regulation. Does post-transcriptional regulation play a role in modulating TNF-α expression in *T. gondii*-infected human macrophages? To answer this question, we focused on the most abundant internal mRNA modification, *N*^6^-methyladenosine (m^6^A), which has been reported as an important mechanism of post-transcriptional regulation [12]. m^6^A is an essential epitranscriptomic mark. Deposition of m^6^A is installed by a large protein complex called methyltransferases, consisting of METTL3, METTL14 and WTAP [13]. Demethylases, mainly FTO and ALKBH5, could remove existing m^6^A modification from mRNA [14,15]. The combination of methyltransferases and demethylases makes m^6^A modification a dynamic and reversible process. Finally, m^6^A modifications are directly recognized and bound by YTH-domain family proteins, including YTHDF1, YTHDF2, YTHDF3, YTHDC1 and YTHDC2 [16–18]. YTH-domain family proteins activate the downstream post-transcriptional regulation pathways, including promoting the degradation of mRNA.

In this study, we found that *T. gondii* infection significantly altered mRNA m^6^A modifications in human macrophages and increased the abundance of m^6^A demethylase FTO. Interestingly, there are m^6^A modification sites on human *TNF-α* mRNA. *T. gondii* could govern the expression of FTO to down-regulate m^6^A modification in the 3’UTR and 5’UTR regions of *TNF-α* mRNA, thereby mounting the TNF-α production in human macrophages. We believe that what we have learned about m^6^A and *T. gondii*-induced TNF-α production will provide important insights into the pathogenesis of toxoplasmosis and the *T. gondii* host-pathogen relationship.

## Materials and methods

### Ethics statement

This study was approved by the Ethics Review Board of Nanjing Medical University [permit no.: NJMU/ (2018) 498].

### Study design

This study aimed to investigate the role of m^6^A modification in regulating the anti-infection immune response of human macrophages against *T. gondii*. Human THP-1 macrophages were infected with tachyzoites of *T. gondii* RH strain to establish an *in vitro* infection model. Uninfected cells served as controls. qPCR was performed to measure the mRNA abundance of inflammation-related genes. The secretion of inflammatory cytokines, including TNF-α, IL-1β, and IL-6, were measured by ELISA. The expression of m^6^A regulators was detected at the mRNA and protein levels using qPCR and Western blotting. RNA-seq was conducted to identify differentially expressed genes in *T. gondii*-infected macrophages compared to controls. m^6^A-seq was used to profile m^6^A modifications in mRNA transcripts. The m^6^A-IP-qPCR assay was performed to validate m^6^A modification levels in *TNF* mRNA. FTO was knocked down in THP-1 macrophages using lentiviral shRNA. Scramble shRNA served as the negative control. Infected FTO-knockdown cells were analyzed for m^6^A modification levels in *TNF* transcripts, expression of inflammation-related genes, secretion of inflammatory cytokines, and *T. gondii* proliferation. To investigate the role of m^6^A readers, RIP assay was performed to detect whether YTHDF1 or YTHDF2 had a direct effect on *TNF-α* mRNA.

### Cell culture

THP-1 cells (human monocytic cell line) and HFF cells (human foreskin fibroblast cell line) were cultured in 1640 medium (Gibco BLR, MD, USA) and Dulbecco’s modified Eagle medium (Gibco BLR, MD, USA), respectively. Both media were supplemented with 10% fetal bovine serum (Gemini, CA, USA) and 1% 100 × penicillin and streptomycin (Gibco BLR, MD, USA). To induce differentiation into a macrophage phenotype, THP-1 cells were treated with 10 ng/ml phorbol myristate acetate (PMA, Sigma-Aldrich) for 72 h. For infection assay, THP-1 macrophages were infected with *T. gondii* RH tachyzoites and GFP-RH tachyzoites at a 1:1 ratio.

### Preparation of *T. gondii* tachyzoites

*T. gondii* RH tachyzoites and GFP-expressing RH tachyzoites were serially subcultured in HFF cell lines. After observing 80% tachyzoite release, cell supernatants were filtered through a 3 μm pore size track-etched membrane (Shanghai Nengthink Filtration Technology Co. Ltd., Shanghai, China) to collect *T. gondii* RH tachyzoites and GFP-RH tachyzoites.

### Real-time qRT-PCR

Total RNA was extracted from cell line samples and *T. gondii* tachyzoites using TRIZOL (Invitrogen, CA, USA). The quality and integrity of the extracted RNA for real-time qRT-PCR and m^6^A-IP-qPCR were evaluated using NanoDrop 2000 (NanoDrop, Wilmington, USA) and Bioanalyzer 2100 (Agilent, CA, USA). Samples with OD260/280 > 1.8, and RNA integrity number (RIN) > 7.0 were then reverse-transcribed to cDNA using HiScript III RT Supermix (Vazyme, Nanjing, China). Semi-quantitative RT-PCR, including cDNA, SYBR Green (Vazyme, Nanjing, China), and 10 μM primer pairs, was performed in a LightCycler 96 Instrument (Roche, Basel, Switzerland). Sequences of gene-specific primers used in this study are provided in S1 Table. Relative expression levels of these genes were calculated using the 2^−(△△Ct)^ method.

### Enzyme-linked immunosorbent assay (ELISA)

After 24 h of *T. gondii* tachyzoites infection, THP-1 macrophage supernatants were harvested. The concentrations of TNF-α, IL-1β, and IL-6 in supernatants were detected using Human TNF-α, IL-1β, and IL-6 ELISA Kits (DaKeWe, Nanjing, China) respectively, following the manufacturer’s instructions.

### Western blotting

For extracting total protein, THP-1 macrophages were lysed in RIPA lysis buffer (Millipore, MA, USA). After centrifugation at 12,000 *g* for 15 min at 4°C, the supernatants of lysates were collected. Protein concentrations were measured using a BCA assay (TIANGEN, Beijing, China). Protein samples were boiled with SDS-PAGE sample loading buffer (Beyotime, Shanghai, China) for 10 min. Appropriate amount of protein sample was resolved on a 10% SDS-PAGE gel, transferred to a 0.2 μm PVDF membrane (Millipore, MA, USA), and then blocked in TBST with 5% non-fat milk. Antibodies included *N*^6^-mA Methyltransferase (METTL3, METTL14, and WTAP) Antibody Sampler Kit (1:1000, Cell Signaling Technology), anti-FTO antibody (1:1000, Abcam), anti-YTHDF1 antibody (1:1000, Proteintech), anti-YTHDF2 antibody (1:1000, Proteintech), and HRP-linked anti-rabbit secondary antibody (1:2000, Cell Signaling Technology). Visualization was performed using ECL luminescence solution (FDbio Science, Hangzhou, China) on a ChemiDoc™ imaging system (Bio-RAD, CA, USA).

### RNA-seq and m^6^A-seq

*T. gondii* RH strain tachyzoites were utilized to infect THP-1 macrophages, forming the infected group, while untreated THP-1 macrophages served as the control group. Total RNA was isolated using TRIzol reagent (Invitrogen, CA, USA). The quality and integrity of the extracted RNA were evaluated by NanoDrop 2000 (NanoDrop) and Bioanalyzer 2100 (Agilent), and were confirmed by agarose gel electrophoresis, as previously reported [19]. Samples with OD260/280 > 1.8, RIN > 7.0, and total amount > 50 μg were processed further. Ribosomal RNA of total RNA was depleted using Epicentre Ribo-Zero Gold Kit (Illumina, CA, USA). Magnesium RNA Fragmentation Module (NEB, MA, USA) was used to fragment RNA into small pieces for 7 min at 86°C. The fragmented RNA was saved for RNA-seq as input control. For m^6^A-seq, the cleaved RNA fragments and m^6^A-specific antibody (Synaptic Systems, Germany) were incubated for 2 h at 4°C in IP buffer. 50 mM Tris-HCl, 750 mM NaCl, and 0.5% Igepal CA-630 were used as IP buffer. Subsequently, the IP RNA was reverse-transcribed to create the cDNA, which were next used to synthesize U-labeled second-stranded DNAs. An A-base is then added to the blunt ends of each strand, preparing them for ligation to the indexed adapters. Then adapters are ligated to the fragments. After the heat-labile UDG enzyme (NEB) treatment of the U-labeled second-stranded DNAs, the ligated products are amplified with PCR by the following conditions: initial denaturation (1 cycle, 95°C for 3 min); amplification (8 cycles, 98°C for 15 s, 60°C for 15 s, and 72°C for 30 s); and then final extension (1 cycle, 72°C for 5 min). Finally, paired-end sequencing (PE150) was performed on an Illumina Novaseq™ 6000, and the data were analyzed by LC-Bio Technology CO., Ltd. (Hangzhou, China). All the sequencing experiments were performed with two biological replicates. The raw sequencing data have been deposited in the Gene Expression Omnibus (GEO) database under accession number GSE288205. Raw reads obtained from the sequencing machine include reads containing adapters and low quality bases which will affect the following bioinformatics analysis. Thus, to get high quality clean reads, reads were further filtered by fastp (https://github.com/OpenGene/fastp, v0.19.4) [20]. For RNA-seq, genes with |log_2_Fold change| ≥ 1 and *P* < 0.05 were defined as differentially expressed genes (DEGs). DEGs were used to perform KEGG pathway enrichment analyses. For m^6^A-seq, DEGs with significantly altered m^6^A modification [false discovery rate (FDR) < 0.05] after *T. gondii* infection were used to perform KEGG pathway enrichment analyses.

### m^6^A-IP qPCR

Firstly, total RNA was isolated using TRIZOL (Invitrogen, CA, USA) and its concentration was adjusted to 1 μg/μl. Subsequently, total RNA was fragmented for 5 min at 94°C in Veriti 96-Well Thermal Cycler (Thermo Fisher Scientific, MA, USA). 1 μg fragmented RNA was reserved as input control. The immunoprecipitation (IP) mixture was incubated for 2 h at 4°C, including m^6^A-specific antibody (Abcam) or mouse IgG (Beyotime), RnaseOUT (Invitrogen), Ribonucleoside vanadyl complexes (RVC, Sigma), and fragmented RNA. Meanwhile, Protein A/G Mix Magnetic Beads (Millipore, MA, USA) was blocked with BSA (Beyotime, Shanghai, China). Finally, magnetic beads were added into the incubated IP mixture for another 2 h at 4°C. The RNA in the magnetic beads was eluted twice and precipitated for further qRT-PCR detection.

### FTO shRNA knock-down assay

Lentiviral shRNA was used to knock down of FTO in THP-1 cells. shRNA targeting sequences are shown below. Human FTO (shFTO): 5’-GGATGACTCTCATCTCGAAGG-3’. Scramble control sequence (NC): 5’-TTCTCCGAACGTGTCACGTAA-3’. Lentiviral particles harboring shRNA targeting FTO (shFTO) and negative control shRNA (NC) were designed and constructed by HANBIO (Shanghai, China). THP-1 cells were cultured in 6-well culture plates using Opti-MEM medium. Then cells were incubated in lentivirus-containing medium with polybrene (Sigma-Aldrich), and selected with puromycin (Solarbio, Beijing, China). The multiplicity of infection (MOI) of lentivirus was 60. FTO knock-down efficiency was assessed via qRT-PCR and Western blotting.

### RNA-binding protein immunoprecipitation (RIP) assay

Anti-YTHDF1 antibody (Proteintech) or anti-YTHDF2 antibody (Proteintech) and Protein A/G Mix Magnetic Beads (Millipore) were pre-incubated overnight at 4°C. 20% percent of cell extracts were taken out as input control. Remaining cell lysates were incubated with previous magnetic bead-antibody complex for 4 h. After elution, RNA was isolated from IP fractions and input control for further detection.

### Statistical analysis

Data from ELISA and qPCR assay was analyzed using Graphpad Prism software (GraphPad). Student’s *t*-test was used for comparisons between two groups. The *P* value < 0.05 was considered statistically significant.

## Results

### *T. gondii* infection upregulated inflammatory cytokine expression in human macrophages

**Fig 1:**
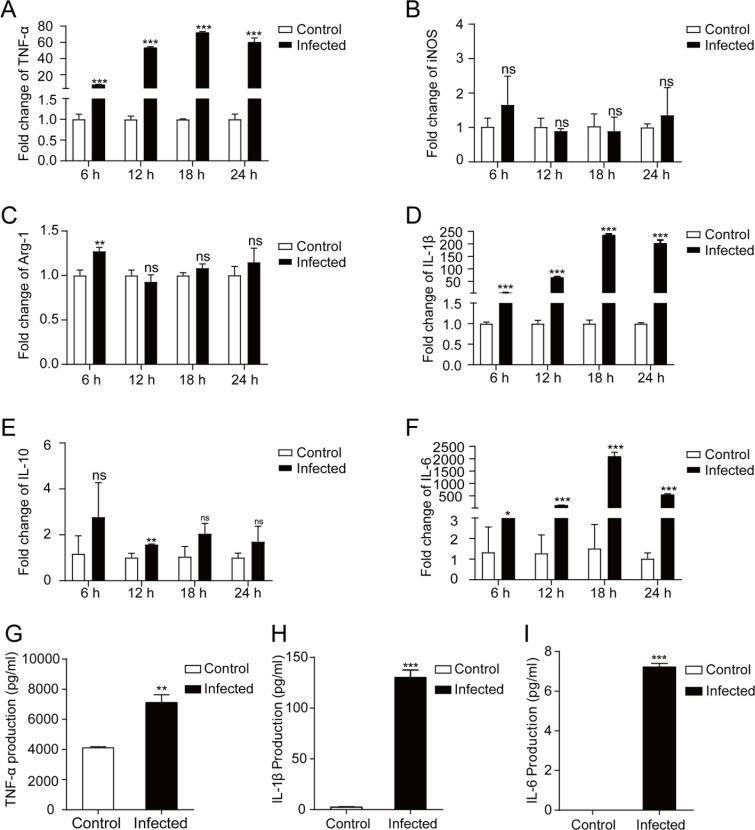
mRNA abundance of inflammatory-related genes and secretion of inflammatory cytokines in THP-1 macrophages infected with *T. gondii* tachyzoites. **A-F:** Transcriptional expression of inflammatory-related genes in THP-1 macrophages after *T. gondii* infection. mRNA abundance of *TNF-α* (A), *iNOS* (B)*, Arg-1* (C)*, IL-1β* (D)*, IL-10* (E), and *IL-6* (F) were quantitatively measured via qRT-PCR. Results are shown as fold changes compared with the control group. Data are represented as the mean ± SD (*n *= 3). Statistical significance was determined using Student’s *t*-test. ns, *P *> 0.05; *, *P *< 0.05; **, *P *< 0.01; ***, *P *< 0.001. **G-I:** Secretion of inflammatory cytokines in THP-1 macrophages after *T. gondii* infection. The secretion of TNF-α (G), IL-1β (H) and IL-6 (I) were detected by ELISA. Results are shown as fold changes compared with the control group. Data are represented as the mean ± SD (*n *= 3). Comparisons between two groups were made by Student’s *t*-test. **, *P *< 0.01; ***, *P *< 0.001.

During *T. gondii* invasion, *T. gondii* antigens interact with host immune cells to trigger the immune responses [21]. Therefore, to analyze the impact of *T. gondii* infection on human macrophages, we used *T. gondii* tachyzoites from RH strain to infect THP-1 macrophages. Inflammatory-related gene expression and the secretion of inflammatory cytokines were measured at 6 h, 12 h, 18 h and 24 h after infection (Fig 1).

The mRNA abundance of *TNF-α*, *IL-1β* and *IL-6* were significantly up-regulated from 6 h to 24 h post infection (Fig 1A, 1D, and 1F), while those of *iNOS*, *Arg-1* and *IL-10* did not change significantly from 18 h to 24 h post infection (Fig 1B, 1C, and 1E). The up-regulation amplitude was *IL-6* > *IL-1β* > *TNF-α*, and the up-regulation amplitude increased with extended infection time within 18 h. Protein abundance of TNF-α, IL-1β, and IL-6 increased at 24 h after infection (Fig 1G, 1H, and 1I), consistent with mRNA abundance. Interestingly, unlike mRNA abundance, protein abundance of TNF-α exhibited the largest increase after *T. gondii* infection (Fig 1G). In sum, the results showed that *T. gondii* tachyzoites could induce the expression of inflammatory-related genes and promote the secretion of inflammatory cytokines, including TNF-α, IL-1β and IL-6.

### *T. gondii* infection increased the expression of m^6^A methyltransferase WTAP and demethylase FTO

**Fig 2:**
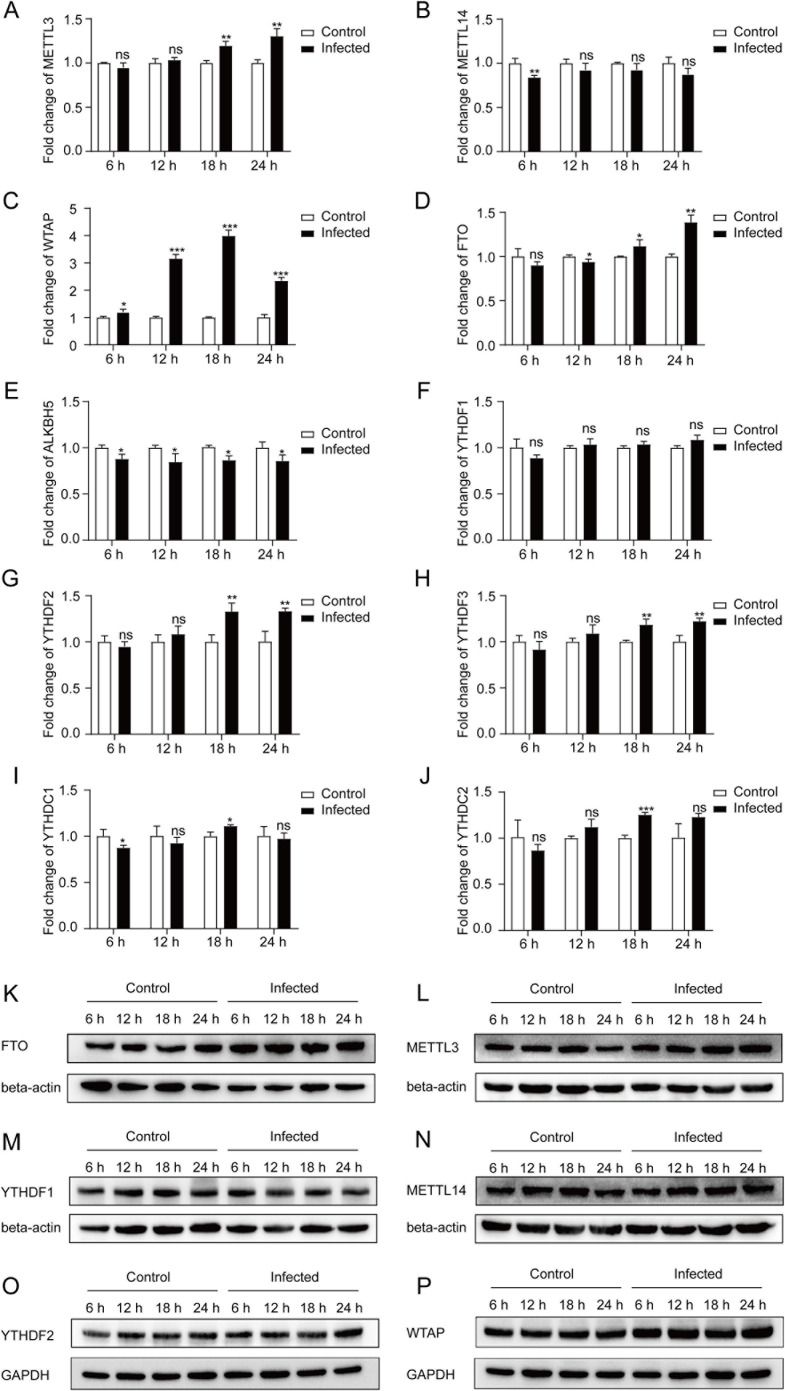
mRNA and protein abundance of m^6^A methyltransferases, demethylases and m^6^A-specific binding proteins in THP-1 macrophages infected with *T. gondii* tachyzoites. **A-J:** ^6^A methyltransferases, m^6^A demethylases and m^6^A-specific binding proteins in THP-1 macrophages after *T. gondii* infection. mRNA abundance of *METTL3* (A), *METTL14* (B), *WTAP* (C), *FTO* (D), *ALKBH5* (E), *YTHDF1* (F), *YTHDF2* (G), *YTHDF3* (H), *YTHDC1* (I), and *YTHDC2* (J) were quantitatively measured by qRT-PCR. Results are shown as fold changes compared with the control group. Data are represented as the mean ± SD (*n *= 3). Comparisons between two groups were made by Student’s *t*-test. ns, *P *> 0.05; *, *P *< 0.05; **, *P *< 0.01; ***, *P *< 0.001. **K-P:** Western blotting analysis for m^6^A methyltransferases, m^6^A demethylases, and m^6^A-specific binding proteins. Protein abundance of FTO (K), METTL3 (L), YTHDF1 (M), METTL14 (N), YTHDF2 (O), and WTAP (P) were measured by Western blotting.

In the regulation of host immune response against pathogens, post-transcriptional regulation responds more rapidly than transcriptional regulation. RNA m^6^A modification plays an important role in post-transcriptional regulation in various pathogen infection models [22–25]. After *T. gondii* infection, the transcriptional expression of methyltransferases, demethylases and m^6^A-specific binding proteins (m^6^A reader proteins) were detected by qRT-PCR (Fig 2A to 2J). At different time points post-infection from 0 h to 24 h, WTAP was highly up-regulated at the transcriptional level, accompanied by a slight decrease in demethylase ALKBH5 (Fig 2C and 2E). Meanwhile, the m^6^A demethylase FTO also showed an upward trend from 18 h to 24 h after *T. gondii* infection (Fig 2D). The mRNA abundance of methyltransferase METTL3, and m^6^A-specific binding protein YTHDF2 and YTHDF3 significantly increased from 18 h to 24 h after infection (Fig 2A, 2G, and 2H).

Then, the protein abundance of m^6^A methyltransferases, demethylases and m^6^A-specific binding proteins were measured at 6 h, 12 h, 18 h, and 24 h after infection. The results showed a significant increase in WTAP and FTO compared to the control group (Fig 2K and 2P). As for METTL3, METTL14, YTHDF1, and YTHDF2, *T. gondii* infection causes slight or no significant changes in protein abundance (Fig 2L-2O).

### *T. gondii* infection affected TNF signaling in human macrophages

**Fig 3:**
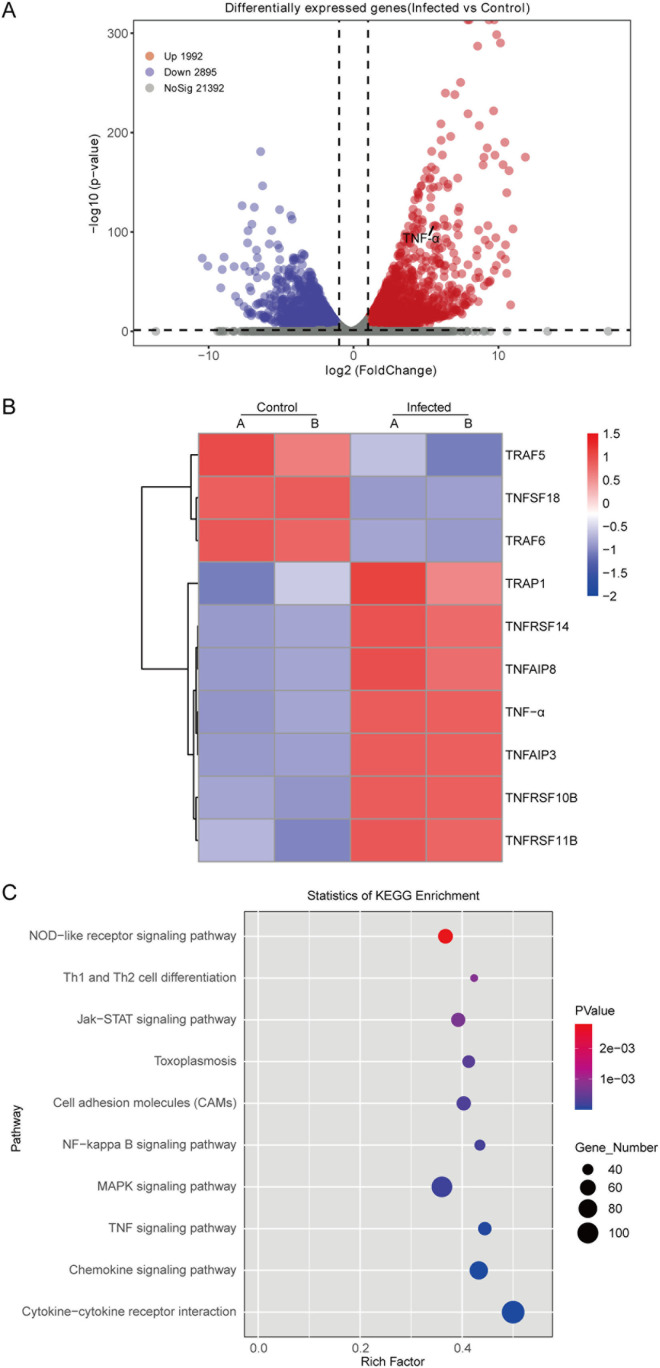
Significantly altered genes and pathways of macrophages after *T. gondii* infection. **A:** Volcano plot of differentially expressed genes. The red dots indicate up-regulated DEGs after *T. gondii* infection. The blue dots indicate down-regulated DEGs after *T. gondii* infection. The gray dots indicate genes without any significant change. Genes with |log_2_Fold change| ≥ 1 and *P* < 0.05 are defined as DEGs. **B:** Heatmap of DEGs related to TNF pathway after *T. gondii* infection. The blue color indicates down-regulated genes. The red color indicates up-regulated genes. **C:** KEGG pathway enrichment analysis of DEGs. The size of the dot is proportional to the number of enriched DEGs, and the rich factor indicates the degree of enrichment.

To thoroughly evaluate the effects of *T. gondii* infection on THP-1 macrophages, we performed global gene expression profiling using RNA-seq. |log_2_Fold change| ≥ 1 and *P* < 0.05 were used as criteria to identify DEGs (differentially expressed genes) between the infected group and the control group. After 24 h-infection of *T. gondii* in THP-1 macrophages, a total of 4887 DEGs were screened, including 1992 up-regulated genes and 2895 down-regulated genes (Fig 3A). Clustering analysis indicated a significant upregulation of *TNF-α* expression level after *T. gondii* infection (Fig 3B). Additionally, the expression of several members of the TNF super-family, including *TRAP1*, *TNFAIP3*, and *TNFAIP8*, were notably altered. KEGG pathway enrichment analysis showed that DEGs were enriched in TNF signaling pathway, toxoplasmosis pathway, cytokine-cytokine receptor interaction pathway, NF-κB signaling pathway, and so on (Fig 3C). These results suggest that *T. gondii* infection could induce an immune response in human macrophages. Proinflammatory cytokine TNF-α significantly changed after infection.

### *T. gondii* infection influenced RNA m^6^A modification in human macrophages

**Fig 4:**
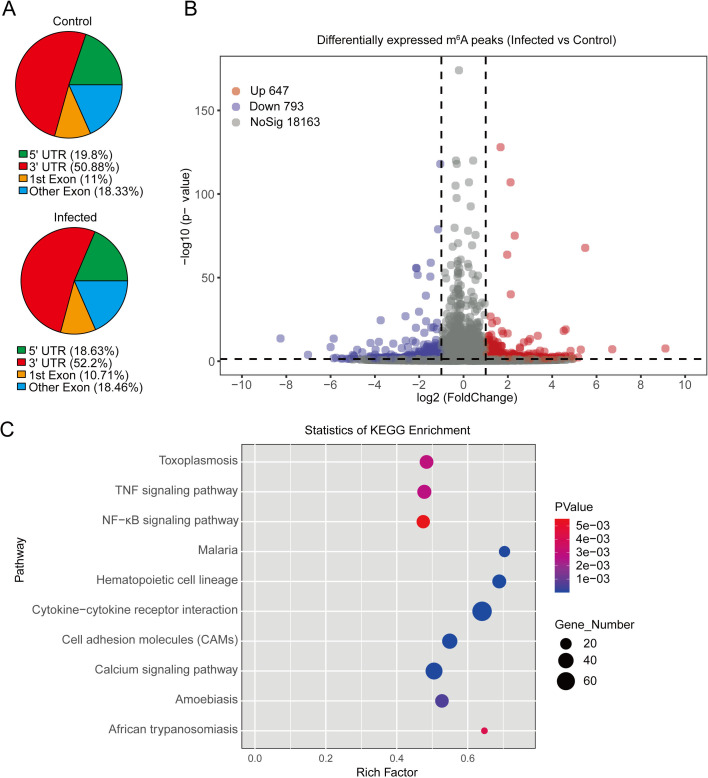
Transcriptome-wide m^6^A methylation profiling of THP-1 macrophages by m^6^A-seq after *T. gondii* infection. **A:** Distribution of m^6^A peaks across the transcriptome of THP-1 macrophages. **B:** Volcano plot of differentially methylated m^6^A peaks. The red dots indicate significantly up-regulated m^6^A methylation after *T. gondii* infection (log_2_Fold change ≥ 1 and *P* < 0.05). The blue dots indicate significantly down-regulated m^6^A methylation after *T. gondii* infection (log_2_Fold change ≤ -1 and *P* < 0.05). The gray dots indicate m^6^A methylation without any significant changes. **C:** KEGG pathway enrichment analysis of DEGs with significantly altered m^6^A modification (FDR < 0.05) after *T. gondii* infection. The size of the dot is proportional to the number of enriched DEGs, and the rich factor indicates the degree of enrichment.

To systematically analyze changes in RNA m^6^A modification in *T. gondii*-infected THP-1 macrophages, we performed m^6^A-seq. After 24 h-infection of *T. gondii* in THP-1 macrophages, m^6^A peaks were mainly distributed in the 3’ UTR and the 5’ UTR regions in both control and infected groups (Fig 4A). *T. gondii* infection increased methylation in the 3’ UTR region, while methylation in the 5’ UTR region decreased. Differentially methylated m^6^A peaks (|log_2_Fold change| ≥ 1 and *P* < 0.05) were identified, with 1440 total (647 up-regulated and 793 down-regulated) in the infected group compared to the control group (Fig 4B). KEGG pathway enrichment analysis of DEGs with significantly altered m^6^A modification (FDR < 0.05) after *T. gondii* infection revealed that such genes were enriched in toxoplasmosis pathway, TNF signaling pathway, NF-κB signaling pathway, and so on (Fig 4C).

### *T. gondii* infection downregulated the m^6^A modification in the 5’UTR and 3’UTR region of *TNF-α* mRNA in human macrophages

**Fig 5:**
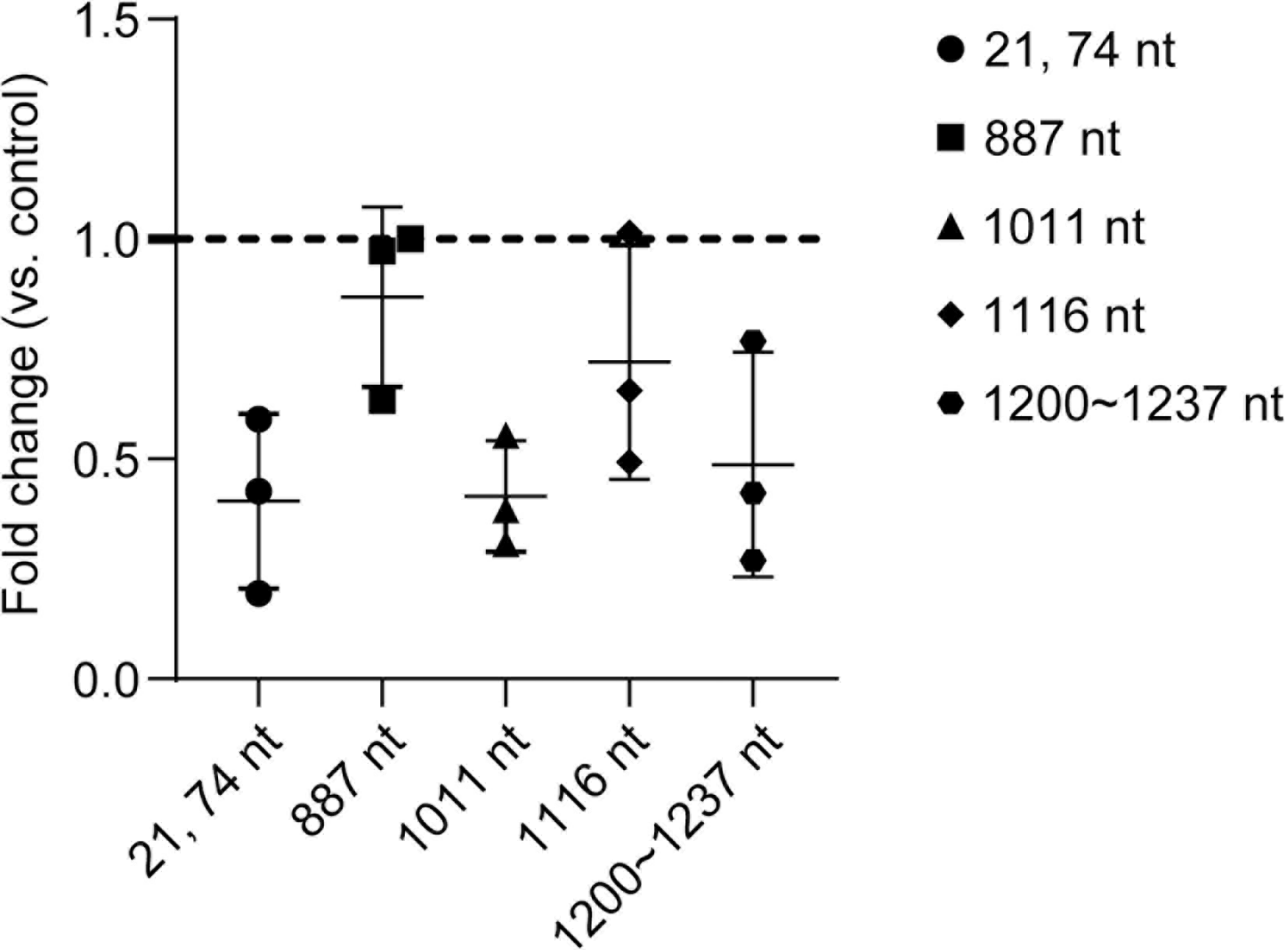
Detection of *TNF-α* mRNA m^6^ A sites after *T. gondii* infection. The m^6^A modification sites on *TNF-α* mRNA were detected by m^6^A-IP-qPCR. Fold change = 1 indicates no change (*n* = 3).

The m^6^A modification is not uniformly distributed in different mRNAs. Given the importance of TNF-α in responding to *T. gondii* infection, we probed the m^6^A modification sites on *TNF-α* mRNA. Firstly, the online prediction tool SRAMP was used to predict the potential m^6^A modification sites on human *TNF-α* mRNA. The results showed that there were 9 potential m^6^A modification sites in the 5’UTR and 3’UTR regions of *TNF-α* mRNA (S2 Table). The m^6^A-seq results suggested that m^6^A modifications in the 5’UTR and 3’UTR region of *TNF-α* mRNA decreased after *T. gondii* infection. Furthermore, we used m^6^A-IP-qPCR to verify the above findings. The m^6^A modification levels decreased at sites both in the 5’UTR region and in the 3’UTR region after *T. gondii* infection (Fig 5).

### Knock-down of FTO attenuated *TNF-α* expression and led to uncontrolled proliferation of *T. gondii* in human macrophages

**Fig 6:**
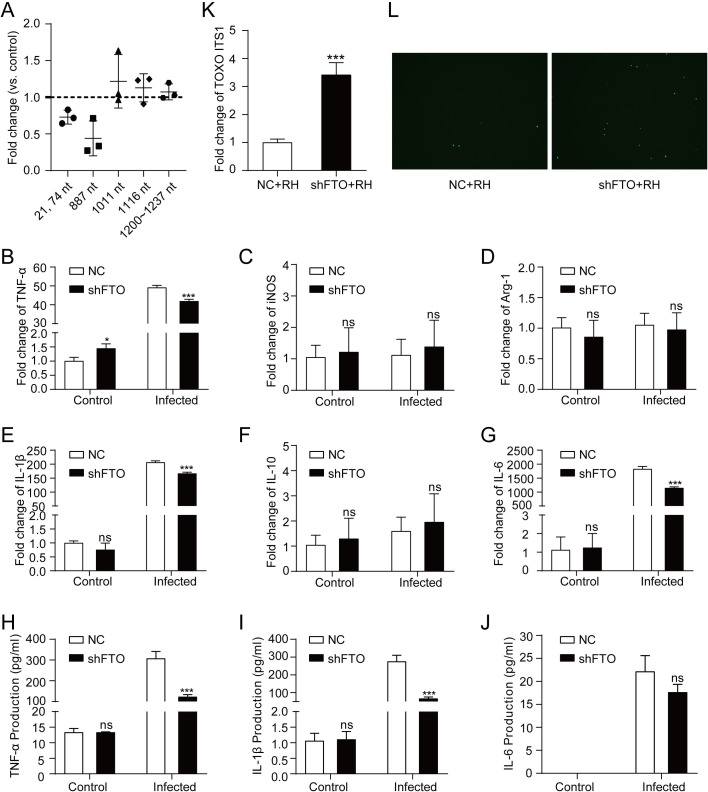
Effects of FTO knock-down on THP-1 macrophage immune response induced by *T. gondii.* A: Changes of m^6^A modification on *TNF-α* mRNA in FTO knock-down cell lines after *T. gondii* infection. Results are expressed as fold change relative to the control group. Fold change = 1 indicates no change (*n* = 3). **B-G:** mRNA abundance of inflammatory-related genes, including *TNF-α* (B), *iNOS* (C), *Arg-1* (D), *IL-1β* (E), *IL-10* (F), and *IL-6* (G), were quantitatively measured via qRT-PCR. Results were shown as fold changes compared with the control group. Data were expressed as the mean ± SD (*n* = 3). Comparisons between two groups were made by Student’s *t*-test. *ns*, *P *> 0.05; *, *P *< 0.05; ***, *P *< 0.001. **H-J:** Secretion of TNF-α (H), IL-1β (I), and IL-6 (J) were detected by ELISA. Results were shown as fold changes compared with the control group. Data were expressed as the mean ± SD (*n* = 3). Comparisons between two groups were made by Student’s *t*-test. *ns*, *P *> 0.05; ***, *P *< 0.001. **K:** The mRNA abundance of *TOXO ITS1* was measured by qRT-PCR. **L:** The prolifera*t*ion of *T. gondii* GFP-expressing tachyzoites was observed by fluorescence microscope.

FTO, a key m^6^A demethylase, was upregulated in THP-1 macrophages after *T. gondii* infection. To investigate the function of FTO in *T. gondii*-induced immune response in macrophages, we knocked down FTO in THP-1 macrophages by shRNA (S1 Fig). In FTO knock-down cell lines, m^6^A modification levels at sites in the 5’UTR and 3’UTR regions of *TNF-α* mRNA did not decrease significantly after *T. gondii* infection (Fig 6A), while *T. gondii* infection down-regulated the m^6^A modification levels at sites in the 5’UTR and 3’UTR regions of *TNF-α* mRNA in wildtype cells (Fig 5). These results indicate that FTO plays a role in *T. gondii*-induced down-regulation of m^6^A modifications in *TNF-α* mRNA.

In the shFTO group, mRNA abundance of *TNF-α*, *IL-1β*, and *IL-6* was significantly lower than that in the control group, while the transcriptional levels of *iNOS*, *Arg-1*, and *IL-10* remained unchanged (Fig 6B-6G). The levels of secreted cytokines after *T. gondii* infection decreased significantly for TNF-α and IL-1β in the shFTO group compared to the control group, and the secretion of IL-6 in the shFTO group showed a decreasing trend but was not statistically significant (Fig 6H-6J). FTO knock-down weakened the immune response induced by *T. gondii*, as evidenced by decreased expression of inflammation-related genes and reduced secretion of inflammatory cytokines.

Additionally, after *T. gondii* infection, the mRNA levels of *TOXO ITS1*, which indicate parasite burden of *T. gondii*, increased significantly in the FTO knock-down group compared with the control group (Fig 6K). Meanwhile, enhanced proliferation of *T. gondii* green fluorescent protein (GFP)-expressing tachyzoites was also observed by the fluorescence microscope in the FTO knock-down group (Fig 6L). These results further demonstrated that FTO knock-down weakened the anti-infection immune response against *T. gondii*.

### *T. gondii* infection inhibited YTHDF2 binding to *TNF-α* mRNA

**Fig 7:**
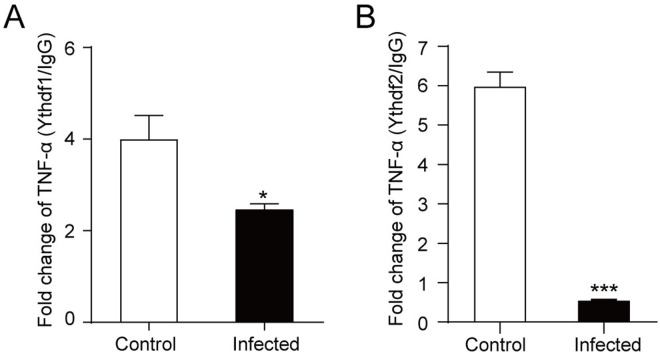
YTHDF1 RIP and YTHDF2 RIP. **A:** qRT-PCR detection of the changes of *TNF-α* enriched by YTHDF1 and IgG. **B:** qRT-PCR detection of the changes of *TNF-α* enriched by YTHDF2 and IgG. Results were shown as fold changes compared with the control group. Data were expressed as the mean ± SD (*n *= 3). Comparisons between two groups were made by Student’s *t*-test. *, *P *< 0.05; ***, *P *< 0.001.

YTHDF proteins are known to bind m^6^A sites and could promote the decay of m^6^A-modified mRNA [26]. An RNA-binding protein immunoprecipitation (RIP) assay was performed to detect whether YTHDF1 or YTHDF2 had a direct effect on *TNF-α* mRNA. After *T. gondii* infection, decreased binding to *TNF-α* mRNA was observed for YTHDF1 (about 2-fold, *P *< 0.05, Fig 7A). Notably, the YTHDF2 RIP assay showed that after *T. gondii* infection, the binding of YTHDF2 to *TNF-α* mRNA significantly down-regulated (about 11-fold, *P *< 0.001), indicating *T. gondii* infection remarkably weakened the binding of YTHDF2 to *TNF-α* mRNA (Fig 7B). This could be attributed to the reduced m^6^A modifications on *TNF-α* mRNA after *T. gondii* infection. These findings suggest that *T. gondii* infection may mount TNF-α expression by inhibiting the degradation of *TNF-α* mRNA.

## Discussion

This study focuses on the post-transcriptional regulation mediated by m^6^A modification in human macrophages after *T. gondii* infection, especially the role of m^6^A in post-transcriptional regulation of TNF-α and the effect of *T. gondii* infection on it. In this study, m^6^A-seq and RNA-seq were conducted to systematically observe the changes of RNA m^6^A modification after *T. gondii* infection. Our transcriptome analysis showed that the expression level of TNF-α was significantly up-regulated in *T. gondii*-treated THP-1 macrophages. Further KEGG pathway analysis indicated that DEGs were enriched in TNF signaling pathway and cytokine-cytokine receptor interaction pathway. Based on the m^6^A-seq data, the m^6^A peaks were mainly enriched in the 3’UTR and the 5’UTR regions. In the infected group, the m^6^A methylation in the 3’UTR region was increased, concomitant with decreased methylation in the 5’UTR region, suggesting that the mRNA splicing, stability and translation were affected. In addition, KEGG pathway enrichment analysis of DEGs with significantly altered m^6^A modification showed that DEGs in toxoplasmosis pathway, TNF signaling pathway, NF-κB signaling pathway were highly enriched. All these findings suggest that there is a close relationship between m^6^A modification alterations and *T. gondii*-induced immune responses, especially the pro-inflammatory cytokine TNF-α.

TNF-α biosynthesis is regulated by a variety of complex mechanisms, which mainly occur in gene transcription, mRNA transport, stability and translation, intracellular signal transduction and other stages [27–30]. At the transcriptional level, TNF-α expression is co-regulated by transcription factors, co-regulators, and chromatin modifications [27]. Our research group found that MIC3, which is a *T. gondii* excretory-secretory antigen, could induce the immune response of mouse macrophages through TLR11/MyD88/NF-κB signaling pathway, and initiate the TNF-α production against *T. gondii*. Post-transcriptional regulation is also essential in TNF-α synthesis. Different microRNAs (miRNAs) and AU-rich elements (AREs) mediate direct or indirect modulation of TNF-α, triggering mRNA degradation, translation inhibition, or translation activation of *TNF-α* mRNA [28–30]. Two recent studies have tried to explore the role of m^6^A in TNF-α production [31,32]. In human macrophages, the loss of m^6^A writer components eliminates m^6^A modification of *TNF* transcripts, thereby enhancing mRNA stability and TNF-α production [31]. Conversely, *Tnf-α* mRNA in murine macrophages was not the direct target of the m^6^A modification [32]. Unlike the commonly studied lipopolysaccharide (LPS)-induced inflammatory response, our study utilized *T. gondii* to infect THP-1 macrophages, and focused on the role of the m^6^A demethylase FTO. We found that after *T. gondii* infection, the upregulated expression of FTO attenuated the m^6^A modification of *TNF* transcripts, thereby enhancing the *TNF* mRNA stability and subsequently increasing TNF-α production. Specifically, we found that there were 9 potential m^6^A modification sites on human *TNF-α* mRNA. After *T. gondii* infection, the m^6^A modification levels at sites in the 5’UTR and 3’UTR regions of *TNF-α* mRNA decreased. Meanwhile, *T. gondii* infection significantly up-regulated the expression of the demethylase FTO in human macrophages. In FTO-knockdown cell lines, the m^6^A modification levels at sites in the 5’UTR region, especially in the 3’UTR region of *TNF-α* mRNA, did not significantly decrease after *T. gondii* infection. This suggests that the *T. gondii* infection-induced decrease in the m^6^A modification levels on *TNF-α* mRNA is associated with FTO. After *T. gondii* infection, the binding of *TNF-α* mRNA to the YTH domain family proteins decreased, suggesting that the degradation of *TNF-α* mRNA was reduced, which would result in increased TNF-α production. Our study elucidated, for the first time, the molecular mechanism of TNF-α expression in human macrophages induced by *T. gondii* infection from an m^6^A perspective.

In host-pathogen interactions, m^6^A modification exhibits a rapid onset of action and plays a crucial role in regulating downstream pathways [33,34]. On one hand, m^6^A modification affects pathogen gene expression by regulating the stability and translational efficiency of pathogen-derived mRNAs, thereby helping pathogens to escape host immune recognition [25,35,36]. On the other hand, m^6^A modification influences the expression of immune-related genes, and regulates the activation and differentiation of immune cells, thereby shaping host immune responses [22–24,32,37,38]. For example, after HIV-1 infection, the global m^6^A modification level of T cell transcriptome was significantly increased, and the immune response was subsequently changed [22]. In the case of *T. gondii*, while two recent studies reported the m^6^A methylation profiles of *T. gondii* tachyzoites and bradyzoites [19,39], our study, to the best of our knowledge, is the first to present the m^6^A landscape across the transcriptome of human macrophages after *T. gondii* infection.

Currently, research on m^6^A modification in the field of parasitology is limited to characterizing the m^6^A modification sites and modification levels of the parasites’ own RNA, identifying homologous m^6^A regulatory proteins, and focusing on the regulatory role of m^6^A modification in the parasite life cycle. For example, there are abundant m^6^A modifications on RNA of the bloodstream form and procyclic form of *Trypanosoma brucei* [40]. Subsequent studies reported that the m^6^A localized in the poly(A)-tailed mRNA of the variant surface glycoprotein (VSG) of *Trypanosoma brucei* inhibits RNA degradation by blocking deadenylation, thus ensuring the stability of VSG transcript [41,42]. PfMT-A70, pfYTH1 and pfYTH2 are m^6^A writer or reader proteins encoded by *Plasmodium falciparum* [43]. m^6^A marks are enriched near the 3’-boundary of *T. gondii* transcripts [39]. METTL3 and WTAP are essential in *T. gondii* viability and depletion of these two writer components prevents parasite replication [39]. Regarding m^6^A methylation differences among different *T. gondii* genotypes, pairwise comparisons of the RH strain (Type I), ME49 strain (Type II), and VEG strain (Type III) identified 735, 192, and 615 differentially methylated peaks (DMPs), as well as 172, 41, and 153 differentially methylated genes (DMGs) [19]. Enrichment analyses associated these DMPs and DMGs with cellular components (e.g., Golgi apparatus, plasma membrane) and signaling pathways (e.g., endocytosis, mTOR signaling), suggesting a role for m^6^A in the pathobiology of *T. gondii*.

It is well-known that *T. gondii* infection universally induces Th1 responses during the acute stage of the disease [44,45]. However, different strains of *T. gondii* induce varying types and levels of cytokines and signaling molecules, ultimately leading to distinct infection outcomes [44–46]. For example, after RH strain (type I) infection, macrophages exhibit only low levels of iNOS expression, resulting in negligible NO production; in contrast, following ME49 strain (type II) infection, macrophages express higher levels of iNOS and produce more NO [44]. Moreover, infection with different strains of *T. gondii* can induce macrophages to polarize into different subtypes [47]. It has been reported that *Toxoplasma* polymorphic effectors, such as ROP16 and GRA15, are involved in the regulation of these processes [47,48]. In this study, we investigated the regulatory role of m^6^A in the immune response of macrophages after infection with *T. gondii* RH strain. As different *T. gondii* strains trigger distinct immune responses in macrophages, besides *Toxoplasma* polymorphic effectors, whether m^6^A plays a role in the distinct host immune responses induced by different *T. gondii* strains is interesting and deserves further investigation.

In this study, we used *T. gondii* tachyzoites and human macrophages to investigate the role of m^6^A in innate immunity against infection. This model reflects the immune response of human macrophages in the acute stage of infection. From an m^6^A perspective, our findings reveal the impact of *T. gondii*, and potentially Apicomplexan protozoa, on public health. Notably, our findings are constrained to acute toxoplasmosis. In immunocompetent individuals, *T. gondii* infection is usually asymptomatic, existing in the form of bradyzoites. Therefore, the role of m^6^A in patients in the chronic stage or those with latent infection needs to be further investigated. Additionally, *T. gondii* infection in healthy populations has been linked to an increased risk of certain diseases, such as neuropsychiatric disorders, metabolic diseases, and autoimmune disorders [49–51]. An increasing number of studies have shown associations between m^6^A and these diseases [52–54]. However, whether a causal relationship exists among *T. gondii* infection, m^6^A alteration, and these diseases awaits further study.

In sum, this study provides new insights into the role of m^6^A modification in macrophage immune responses against *T. gondii*. Additionally, our findings uncover a new molecular mechanism for the immediate gene activation of TNF-α, through which m^6^A modification regulates TNF-α expression.

## Supporting information

S1 Table Primers used in this study. (DOCX)

S2 Table Prediction of m^6^A modification sites on human *TNF-α* mRNA. (DOCX)

S1 Fig The efficacy of FTO knock-down. A: qRT-PCR analysis of FTO knock-down efficacy. The mRNA abundance of *FTO* was measured by qRT-PCR. Data are shown as mean ± SD (*n *= 3). Statistical significance was determined using Student’s *t*-test. ***, *P* < 0.001. B: Western blotting analysis of FTO knock-down efficacy.
(TIF)

S1 Text Original images for Western blotting. (DOCX)

S1 Data Original values used to build graphs. (XLSX)
