# Supplementary material for: The m6A demethylase FTO regulates TNF-α expression in human macrophages following Toxoplasma gondii infection
Source: PLoS Negl Trop Dis. 2025 Jul 15;19(7):e0013289. doi: 10.1371/journal.pntd.0013289 (PMC12282902; doi:10.1371/journal.pntd.0013289)
Supplement: S1 Table — (DOCX) [file pntd.0013289.s001.docx]

**S1 Table.** Primers used in this study.

| Name |  | Sequence（5’-3’） |
| --- | --- | --- |
| Homo-GAPDH | Forward primer | TATGACAACAGCCTCAAGAT |
|  | Reverse primer | AGTCCTTCCACGATACCA |
| Homo-TNF-α | Forward primer | GCCTGTACCTCATCTACTC |
|  | Reverse primer | CCTTGGTCTGGTAGGAGA |
| Homo-iNOS | Forward primer | GGAGGTGCTAGAGGAGTT |
|  | Reverse primer | AGGAGCTGATGGAGTAGAA |
| Homo-Arg-1 | Forward primer | GTGTGATGTGAAGGATTATGG |
|  | Reverse primer | TTCTTCCGTTCTTCTTGACT |
| Homo-IL-1β | Forward primer | CGACCACCACTACAGCAAG |
|  | Reverse primer | AAAGATGAAGGGAAAGAAGG |
| Homo-IL-10 | Forward primer | GAAAGGCATCTACAAAGC |
|  | Reverse primer | GTTTCGTATCTTCATTGTCA |
| Homo-IL-6 | Forward primer | AAGTCCTGATCCAGTTCCT |
|  | Reverse primer | GCAGAATGAGATGAGTTGTC |
| Homo-METTL3 | Forward primer | TCGTTAGTCTCTGGTCTGA |
|  | Reverse primer | TCTTGCTCTGTTGTTCCTTA |
| Homo-METTL14 | Forward primer | CAGAAGTTACGGCGACAG |
|  | Reverse primer | GAGCAGAGGTATCATAGGAAG |
| Homo-WTAP | Forward primer | CAACACAACCGAAGATGAC |
|  | Reverse primer | CGTAACCACTACCTCCTCT |
| Homo-FTO | Forward primer | TGCTGTGCCATTGTGTAT |
|  | Reverse primer | GTATGCTGCTCTGCTCTTA |
| Homo-ALKBH5 | Forward primer | GTTCCAGTTCAAGCCTATTC |
|  | Reverse primer | TTCATCAGCAGCATATCCA |
| Homo-YTHDF1 | Forward primer | TCCATACCTCACCACCTAC |
|  | Reverse primer | GAACCTGTGCTGATAGATGT |
| Homo-YTHDF2 | Forward primer | GCAACAGACACAGCCATT |
|  | Reverse primer | TCCTACTCCATTACCATCCA |
| Homo-YTHDF3 | Forward primer | GGTCTCAGGATAAGTGGAAG |
|  | Reverse primer | TCTAAGCGAATATGCCGTAA |
| Homo-YTHDC1 | Forward primer | AGAGGAGGAGGAGGAAGA |
|  | Reverse primer | ACAGATTCAGAACCAGAGTC |
| Homo-YTHDC2 | Forward primer | CGCCTGTCACTATATTGGT |
|  | Reverse primer | CACTATCACTACTGTCATTGG |
| Homo-TNF-α-m^6^A-1 | Forward primer | TCAGCAAGGACAGCAGAG |
|  | Reverse primer | AGTATGTGAGAGGAAGAGAAC |
| Homo-TNF-α-m^6^A-2 | Forward primer | GTGAGGAGGACGAACATC |
|  | Reverse primer | TGAGCCAGAAGAGGTTGA |
| Homo-TNF-α-m^6^A-3 | Forward primer | CAACCTCTTCTGGCTCAA |
|  | Reverse primer | CGAAGTGGTGGTCTTGTT |
| Homo-TNF-α-m^6^A-4 | Forward primer | GCTGGCAACCACTAAGAA |
|  | Reverse primer | GTCTCCAGATTCCAGATGTC |
| Homo-TNF-α-m^6^A-5 | Forward primer | GACATCTGGAATCTGGAGAC |
|  | Reverse primer | GCCTAAGGTCCACTTGTG |
| TOXO ITS1 | Forward primer | CAAGAAGCGTGATAGTATCG |
|  | Reverse primer | CTGAAGAAACTCCTGGAAATC |
