# Supplementary material for: The m6A demethylase FTO regulates TNF-α expression in human macrophages following Toxoplasma gondii infection
Source: PLoS Negl Trop Dis. 2025 Jul 15;19(7):e0013289. doi: 10.1371/journal.pntd.0013289 (PMC12282902; doi:10.1371/journal.pntd.0013289)
Supplement: S2 Table — (DOCX) [file pntd.0013289.s002.docx]

**S2 Table.** Prediction of m^6^A modification sites on human *TNF-α* mRNA.

| No. | Position | Region | m^6^A site prediction score | m^6^A site confidence |
| --- | --- | --- | --- | --- |
| 1 | 21 | 5’UTR | 0.830 | m^6^A site (Very high confidence) |
| 2 | 74 | 5’UTR | 0.737 | m^6^A site (Very high confidence) |
| 3 | 887 | 3’UTR | 0.788 | m^6^A site (Very high confidence) |
| 4 | 1011 | 3’UTR | 0.867 | m^6^A site (Very high confidence) |
| 5 | 1116 | 3’UTR | 0.600 | m^6^A site (Moderate confidence) |
| 6 | 1200 | 3’UTR | 0.897 | m^6^A site (Very high confidence) |
| 7 | 1210 | 3’UTR | 0.793 | m^6^A site (Very high confidence) |
| 8 | 1227 | 3’UTR | 0.795 | m^6^A site (Very high confidence) |
| 9 | 1237 | 3’UTR | 0.836 | m^6^A site (Very high confidence) |
