# Supplementary figures and images for: The m6A demethylase FTO regulates TNF-α expression in human macrophages following Toxoplasma gondii infection
Source: PLoS Negl Trop Dis. 2025 Jul 15;19(7):e0013289. doi: 10.1371/journal.pntd.0013289 (PMC12282902; doi:10.1371/journal.pntd.0013289)

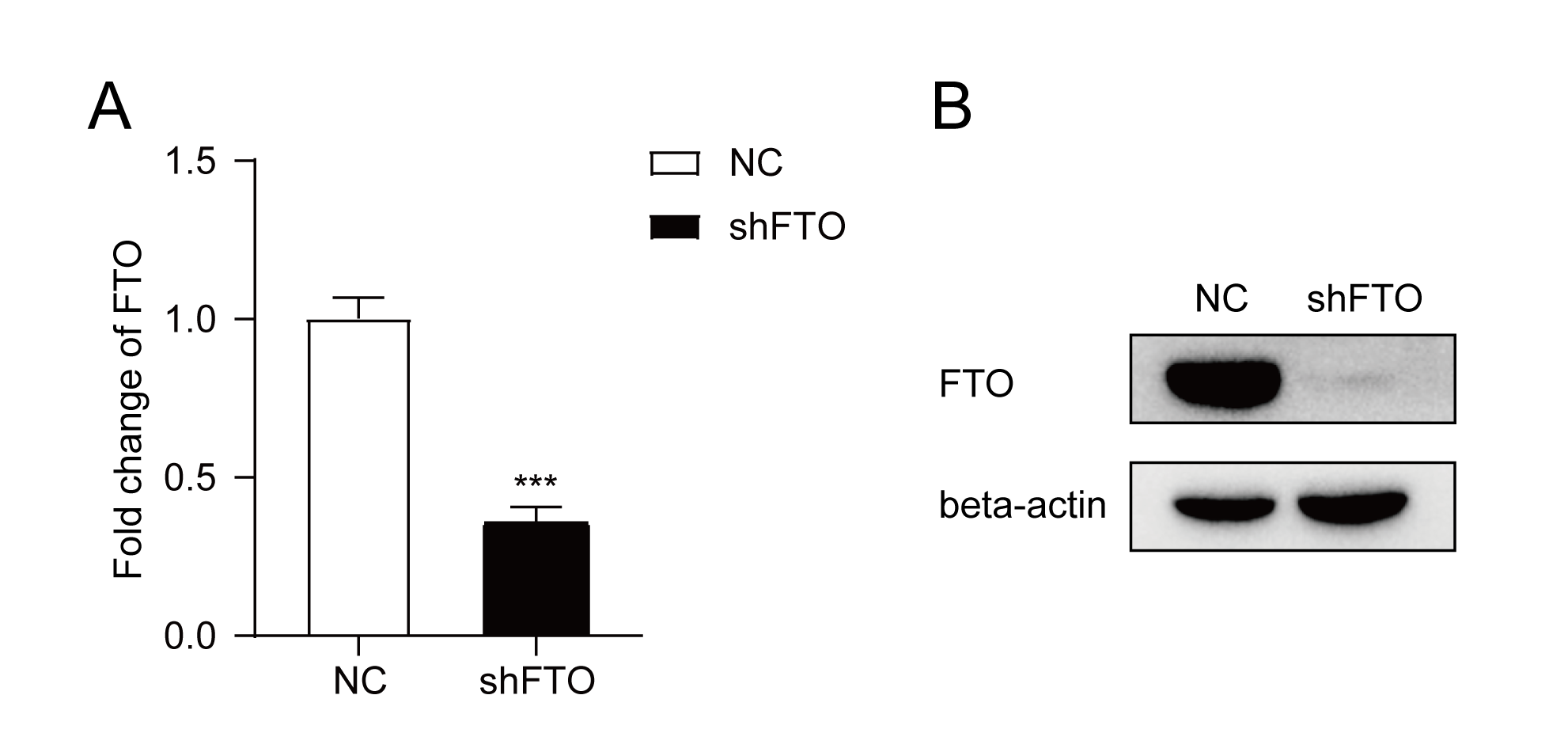

Supplement: S1 Fig — A: qRT-PCR analysis of FTO knock-down efficacy. The mRNA abundance of FTO was measured by qRT-PCR. Data are shown as mean ± SD (n = 3). Statistical significance was determined using Student’s t-test. ***, P < 0.001. B: Western blotting analysis of FTO knock-down efficacy. (TIF) [file pntd.0013289.s003.tif]
