# Supplementary material for: The m6A demethylase FTO regulates TNF-α expression in human macrophages following Toxoplasma gondii infection
Source: PLoS Negl Trop Dis. 2025 Jul 15;19(7):e0013289. doi: 10.1371/journal.pntd.0013289 (PMC12282902; doi:10.1371/journal.pntd.0013289)

**S1 Text. Original images for Western blotting**

**Original images for Fig 2K**

FTO


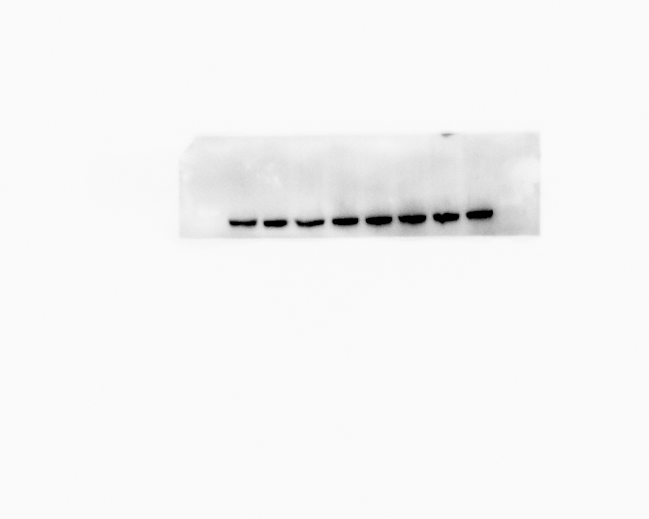


beta-actin


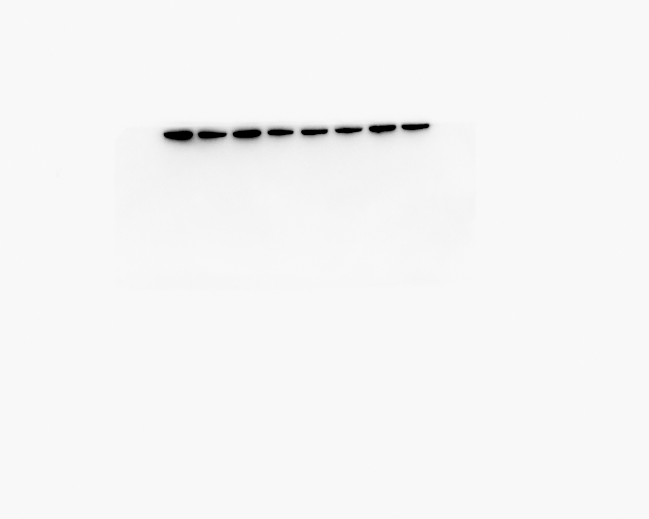


**Original images for Fig 2L**

METTL3


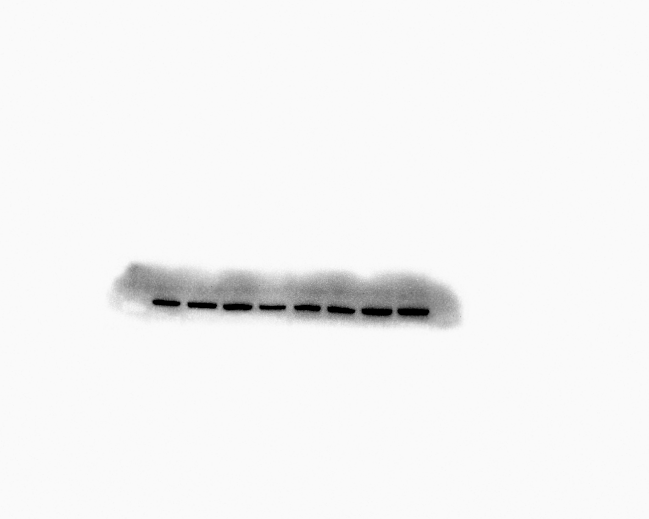


beta-actin


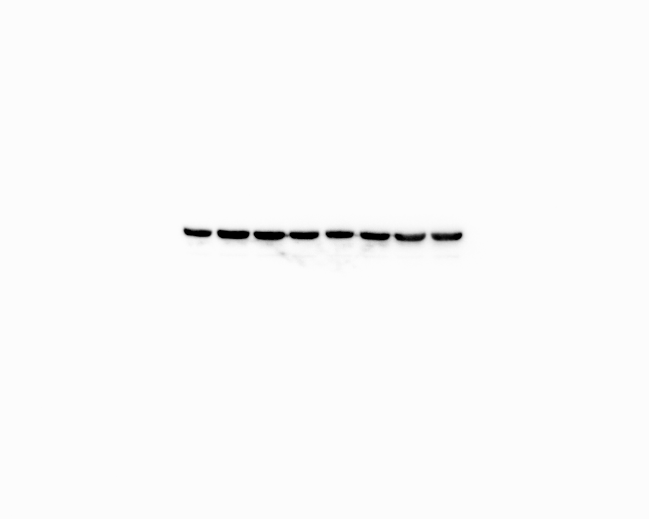


**Original images for Fig 2M**


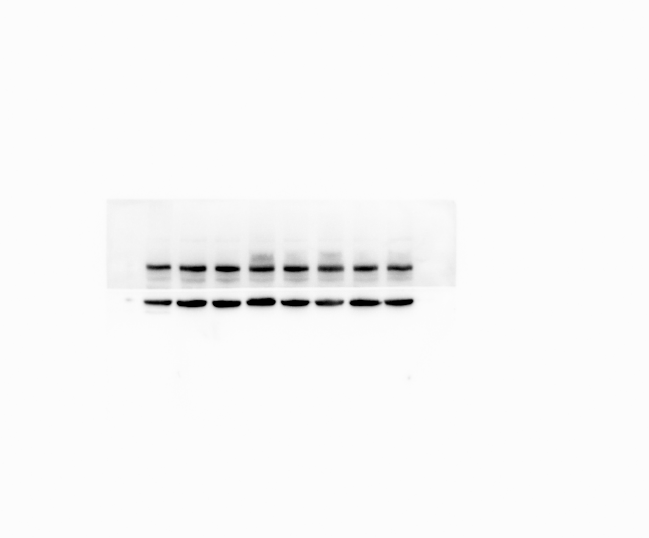


**beta-actin**

**YTHDF1**

**Original images for Fig 2N**

METTL14


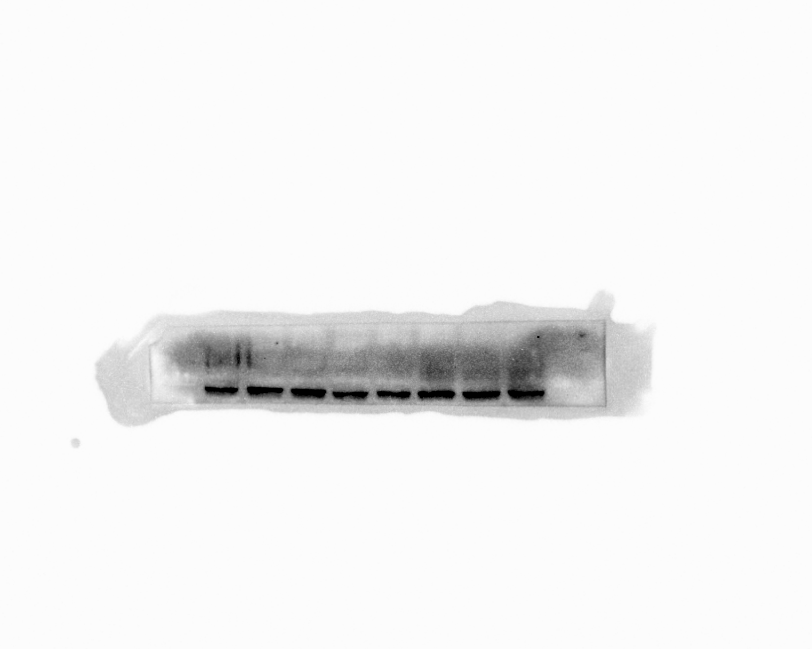


beta-actin


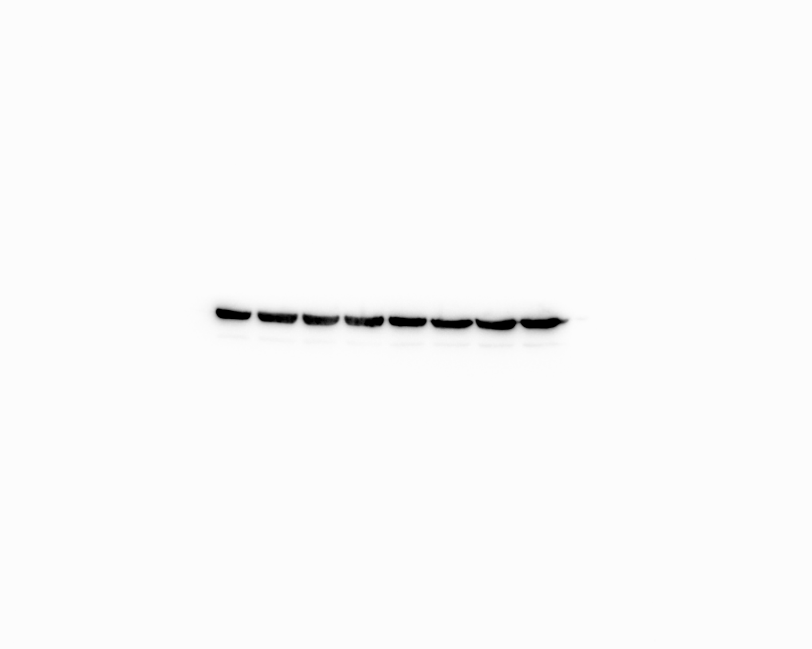


**Original images for Fig 2O**

YTHDF2


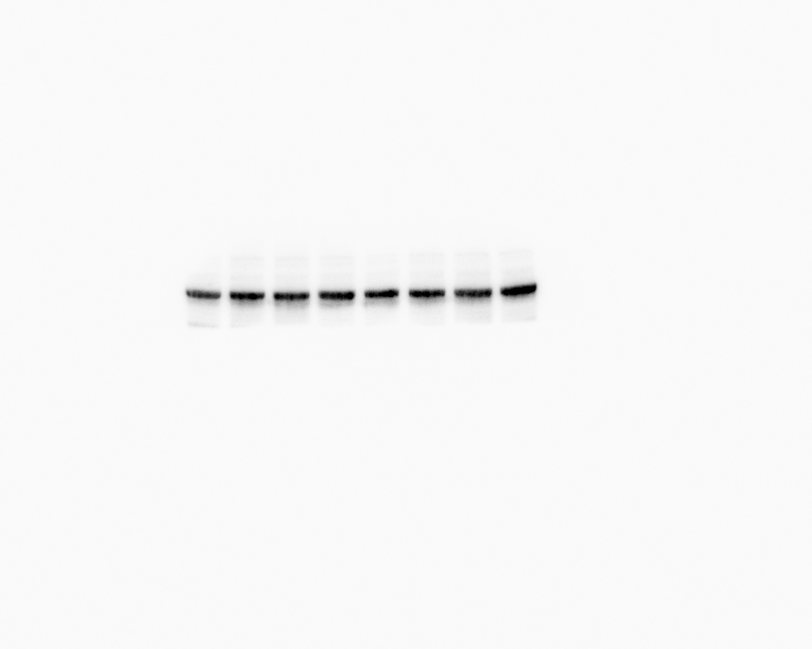


GAPDH


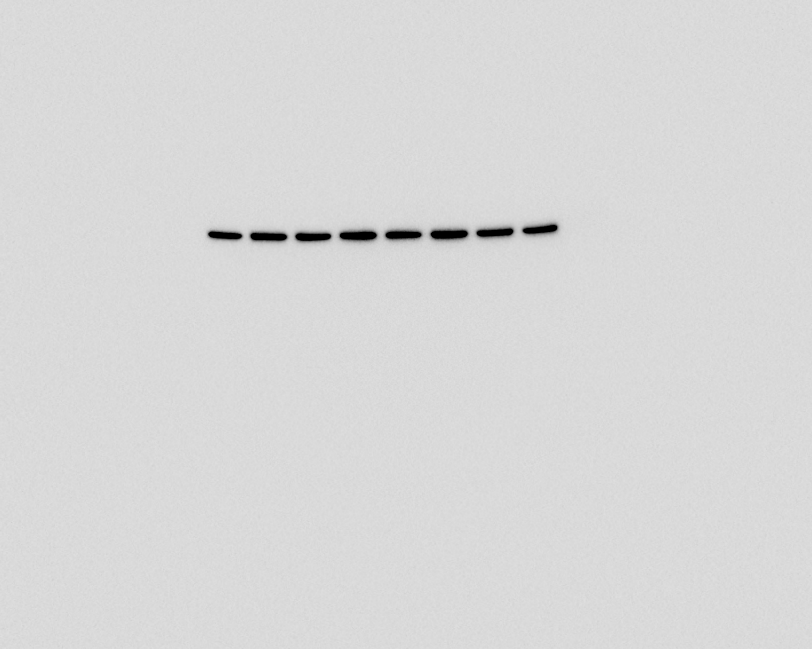


**Original images for Fig 2P**

WTAP


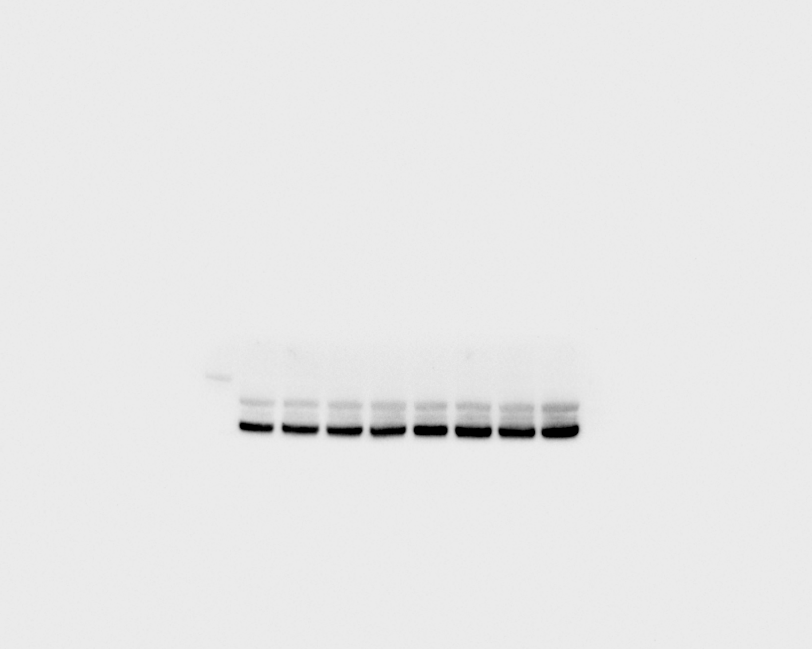


GAPDH


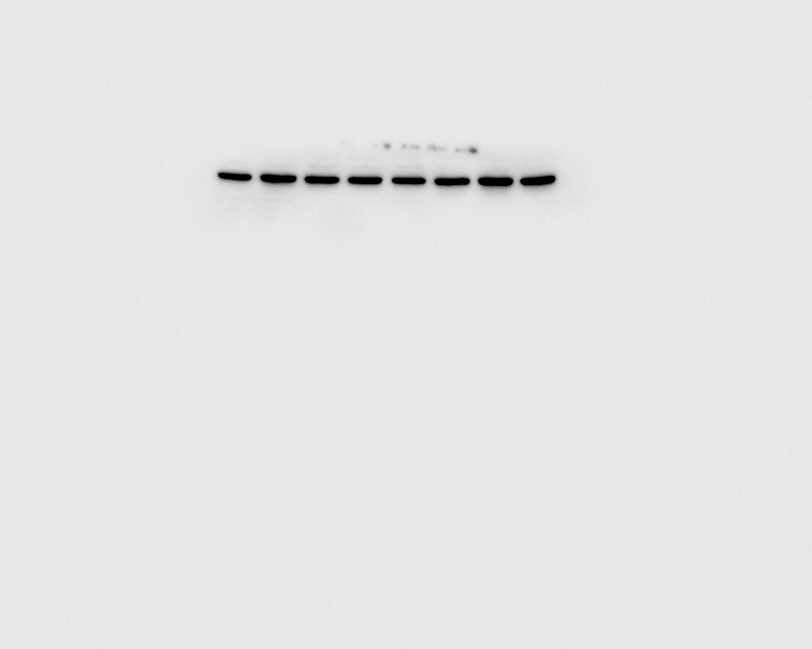


**Original images for Fig S1B**

FTO


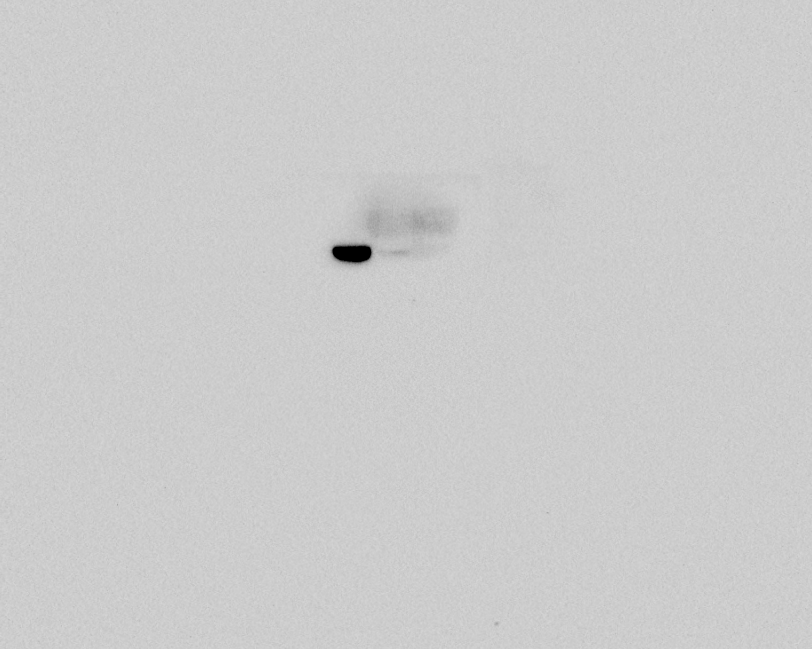


beta-actin


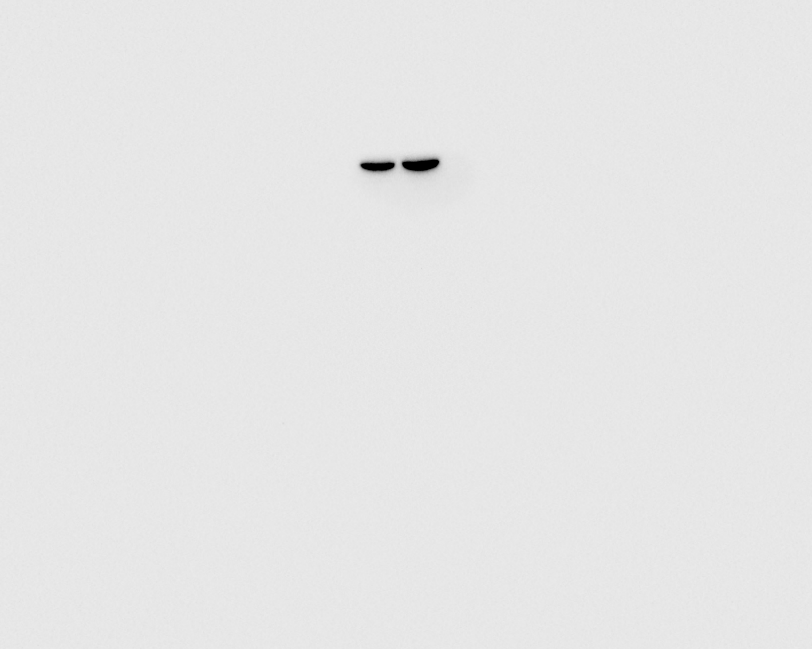

Supplement: S1 Text — (DOCX) [file pntd.0013289.s004.docx]
